# Supplementary material for: Study on crude oil displacement efficiency by fracturing fluid in tight sandstone reservoir
Source: PLoS One. 2025 Nov 4;20(11):e0335233. doi: 10.1371/journal.pone.0335233 (PMC12585015; doi:10.1371/journal.pone.0335233)
Supplement: S1 File — The fundamental principle and detailed derivation process for converting the NMR relaxation time into pore size of core samples through high-pressure mercury intrusion experiments and nuclear magnetic resonance (NMR) testing. (PDF) [file pone.0335233.s001.pdf]

S1 The method for converting T_2 on the X-axis of NMR tests into pore radius

NMR transverse relaxation time (T_2) of fluid in porous media can be determined by the following equation:

$$\frac{1}{T_2} = \frac{1}{T_{2,bulk}} + \frac{1}{T_{2,surface}} + \frac{1}{T_{2,diffusion}} \tag{1}$$

where $T_{2,bulk}$ stands for the bulk relaxation time of the pore-filling fluid (ms), $T_{2,surface}$ represents the surface relaxation time (ms), and $T_{2,diffusion}$ denotes the relaxation time caused by diffusion (ms).

$T_{2,bulk}$ is usually ignored as the amplitude of $T_{2,bulk}$ is notably larger than that of T_2 for fluid flow in porous media. $T_{2,diffusion}$ is also ignored when the magnetic field used is uniform with a quite small field gradient. Therefore, T_2 is dominantly dependent on $T_{2,surface}$, which is corresponded to the specific surface area of a pore. $T_{2,surface}$ can be expressed as follows:

$$T_2 \approx T_{2,surface} = \rho(\frac{S}{V})_{pore} \tag{2}$$

where S stands for the interstitial surface area (μm^2), ρ denotes the surface relaxivity (μm /ms), and V represents the pore volume, PV(μm^3).

And S/V can be rewritten as a function of the dimensionless shape factor of a pore, F_s, and pore radius, r (μm):

$$\frac{S}{V} = \frac{F_s}{r} \tag{3}$$

Integrating Eq. (2) with Eq. (3), $T_{2,surface}$ can be expressed as:

$$T_2 = r/C \tag{4}$$

where $C = \rho F_s$, and C denotes a constant conversion coefficient (ms/μm).
