# Supplementary material for: Study on crude oil displacement efficiency by fracturing fluid in tight sandstone reservoir
Source: PLoS One. 2025 Nov 4;20(11):e0335233. doi: 10.1371/journal.pone.0335233 (PMC12585015; doi:10.1371/journal.pone.0335233)
Supplement: Attachment — Submitted filename: Review-PONE-D-25-35735.docx [file pone.0335233.s003.docx]

This manuscript focuses on the study of the displacement efficiency of fracturing fluid on crude oil in tight sandstone reservoirs, conducting relevant experiments, and exhibiting certain innovativeness and practicality. However, there are some deficiencies need to be checked out and revised:

1. In Introduction section, author mentioned imbibition, but there is a lack of the relevance of imbibition to the main work of this manuscript. It is recommended that author should clarify this relevance to enhanced the readability of the manuscript.
2. The relationship of imbibition and the flowback of fracturing fluid should be clarified in introduction (for example, the flowback of fracturing fluid may be achieved through imbibition).
3. The case of letters in table 1 needs to be consistent.
4. It is recommended that other physical parameters of experimental cores such as length, diameter and porosity should be shown in table 1.
5. Is the method that converting the abscissa of the NMR T₂ spectrum to pore-throat radius commonly used in the industry? Author should appropriately cite some relevant literatures to demonstrate its rationality.
6. Introduction section mentioned that the study revealed the main factors affecting oil-water displacement during the flowback process of tight oil fracturing, but the discussions of main factors in Section 2 and 3 is not enough, which is confused.
7. The manuscript only reviewed the effect of capillary force on imbibition, while gravity also plays an important role in affecting imbibition. To better explain the background and relevant theories of this study, it is recommended to cite the following literatures:
a) <https://doi.org/10.1016/j.geoen.2024.213071>.
b) <https://doi.org/10.2118/224403-PA>.
c) <https://doi.org/10.1080/01932691.2023.2177670>.
d) <https://doi.org/10.1016/j.geoen.2024.213057>.
