# Supplementary material for: Study on crude oil displacement efficiency by fracturing fluid in tight sandstone reservoir
Source: PLoS One. 2025 Nov 4;20(11):e0335233. doi: 10.1371/journal.pone.0335233 (PMC12585015; doi:10.1371/journal.pone.0335233)
Supplement: Attachment — Submitted filename: Detailed Response to Reviewers.docx [file pone.0335233.s005.docx]

**Re: The Itemized Response to Comments Made by Editor**

Journal:	**PLOS One**

Manuscript ID:	**PONE-D-25-35735**

Title:	**Study on crude oil displacement efficiency by fracturing fluid in tight sandstone reservoir**

Dear Academic Editor:

Thank you for your letter and for the comments and requirements concerning our manuscript entitled “Study on crude oil displacement efficiency by fracturing fluid in tight sandstone reservoir” (ID: PONE-D-25-35735). Those comments and requirements are all valuable and very helpful for revising and improving our paper, as well as the important guiding significance to our researches. We have studied comments and requirements carefully and have made correction which we hope meet with approval. Revised portion are marked in red in the paper. The main corrections in the paper and the responds to the comments and requirements are as flowing:

When submitting your revision, we need you to address these additional requirements.

**Comment #1**: Please ensure that your manuscript meets PLOS ONE's style requirements, including those for file naming. The PLOS ONE style templates can be found at <https://journals.plos.org/plosone/s/file?id=wjVg/PLOSOne> formatting sample main body.pdf and <https://journals.plos.org/plosone/s/file?id=ba62/PLOSOne> formatting sample title authors affiliations.pdf.

**Response:** Thank you very much for your reminder regarding the formatting requirements of PLOS ONE. We have carefully revised the manuscript according to the journal’s style guidelines, including file naming conventions, title and author formatting, and manuscript structure.

**Action:** All formatting has been adjusted to meet PLOS ONE’s requirements. The detailed modifications can be found in the revised manuscript (highlighted in red).

**Comment #2**: We note that this submission includes NMR spectroscopy data. We would recommend that you include the following information in your methods section or as Supporting Information files:

1) The make/source of the NMR instrument used in your study, as well as the magnetic field strength. For each individual experiment, please also list: the nucleus being measured; the sample concentration; the solvent in which the sample is dissolved and if solvent signal suppression was used; the reference standard and the temperature.

2) A list of the chemical shifts for all compounds characterized by NMR spectroscopy, specifying, where relevant: the chemical shift (δ), the multiplicity and the coupling constants (in Hz), for the appropriate nuclei used for assignment.

3)The full integrated NMR spectrum, clearly labelled with the compound name and chemical structure.

We also strongly encourage authors to provide primary NMR data files, in particular for new compounds which have not been characterized in the existing literature. Authors should provide the acquisition data, FID files and processing parameters for each experiment, clearly labelled with the compound name and identifier, as well as a structure file for each provided dataset. See our list of recommended repositories here: https://journals.plos.org/plosone/s/recommended-repositories.

**Response:** Thank you very much for your detailed comment on the NMR spectroscopy data. We would like to clarify that the NMR tests conducted in this study are different from conventional NMR spectroscopy used for compound characterization. Specifically, our experiments employed low-field Nuclear Magnetic Resonance (NMR) technology, which is widely used to characterize the distribution of fluids in porous media. The purpose of these tests was to investigate the pore-scale oil displacement characteristics of tight sandstone cores.

In our study, the principle of low-field NMR is as follows: under an external magnetic field, the hydrogen nuclei of crude oil resonate when their oscillation frequency matches the applied frequency, absorbing energy and generating an NMR signal. After the radiofrequency pulse is turned off, the nuclei release energy and gradually return to equilibrium during a relaxation process. The echo attenuation signals collected from oil-saturated cores are then processed using mathematical inversion methods to obtain T_2_ spectra, which provide information about the pore structure and fluid distribution within the cores. According to the journal’s requirements, we have supplemented the detailed information of the NMR instrument (make, source, and magnetic field strength) in the Experimental Section. Additionally, we have provided in the Supporting Information files the detailed principles of the NMR characterization and the original NMR data files of the tested core samples.

**Action:** The following statement was added to Experimental Section:

“In this study, a MesoMR23-060 NMR imaging system (Suzhou Niumag Analytical Instrument Corporation, Suzhou, China) was used for core testing to investigate the distribution of crude oil within the core samples. This equipment is equipped with an independent pulse control module and a radio frequency (RF) transmission/reception circuit, which improves the accuracy and stability of pulse sequences and enhances the signal-to-noise ratio of NMR signals. The spectrometer frequency of the RF pulse used in this study was 21 MHz, and the waiting time between two scans of sampling data was 4000 ms.”

And the basic principles of NMR tests for characterizing the distribution of crude oil within the cores, as well as the original NMR data, are provided in the Supporting Information.

**Comment #3**: We note that your Data Availability Statement is currently as follows: All relevant data are within the manuscript and its Supporting Information files.

Please confirm at this time whether or not your submission contains all raw data required to replicate the results of your study. Authors must share the “minimal data set” for their submission. PLOS defines the minimal data set to consist of the data required to replicate all study findings reported in the article, as well as related metadata and methods (https://journals.plos.org/plosone/s/data-availability#loc-minimal-data-set-definition).

For example, authors should submit the following data:

- The values behind the means, standard deviations and other measures reported;

- The values used to build graphs;

- The points extracted from images for analysis.

Authors do not need to submit their entire data set if only a portion of the data was used in the reported study.

If your submission does not contain these data, please either upload them as Supporting Information files or deposit them to a stable, public repository and provide us with the relevant URLs, DOIs, or accession numbers. For a list of recommended repositories, please see https://journals.plos.org/plosone/s/recommended-repositories.

If there are ethical or legal restrictions on sharing a de-identified data set, please explain them in detail (e.g., data contain potentially sensitive information, data are owned by a third-party organization, etc.) and who has imposed them (e.g., an ethics committee). Please also provide contact information for a data access committee, ethics committee, or other institutional body to which data requests may be sent. If data are owned by a third party, please indicate how others may request data access.

**Response:** We sincerely thank the editor for this helpful reminder. We confirm that all raw data required to replicate the results of this study have been fully provided. The detailed datasets, including the data used to construct the figures, as well as the original NMR data, are all included in the Supporting Information files. These materials ensure that the minimal data set defined by PLOS ONE is openly available for replication and verification.

We greatly appreciate the editor’s careful consideration and constructive guidance.

**Action:** All the original data underlying this study are provided in the Supporting Information files, where the details can be fully accessed.

**Comment #4**: Thank you for stating the following financial disclosure:

This research is supported by the National Natural Science Foundation of China (No.52174031) and the Youth Innovation Team of Shaanxi Universities.

Please state what role the funders took in the study. If the funders had no role, please state: "The funders had no role in study design, data collection and analysis, decision to publish, or preparation of the manuscript."

If this statement is not correct you must amend it as needed.

Please include this amended Role of Funder statement in your cover letter; we will change the online submission form on your behalf.

**Response:** We sincerely thank the editor for the reminder regarding the financial disclosure. We confirm that the funders had no role in study design, data collection and analysis, decision to publish, or preparation of the manuscript.

**Comment #5**: Thank you for stating the following in the Acknowledgments Section of your manuscript: This research is supported by the National Natural Science Foundation of China (No.52174031) and the Youth Innovation Team of Shaanxi Universities.

We note that you have provided funding information that is not currently declared in your Funding Statement. However, funding information should not appear in the Acknowledgments section or other areas of your manuscript. We will only publish funding information present in the Funding Statement section of the online submission form.

Please remove any funding-related text from the manuscript and let us know how you would like to update your Funding Statement. Currently, your Funding Statement reads as follows:

This research is supported by the National Natural Science Foundation of China (No.52174031) and the Youth Innovation Team of Shaanxi Universities.

Please include your amended statements within your cover letter; we will change the online submission form on your behalf.

**Response:** We sincerely thank the editor for the reminder regarding the placement of funding information. We have carefully revised the manuscript and removed all funding-related text from the Acknowledgments section and other parts of the manuscript.

The Funding Statement provided by the editor - “This research is supported by the National Natural Science Foundation of China (No.52174031) and the Youth Innovation Team of Shaanxi Universities.” - is correct. We have also included this Funding Statement in our cover letter as requested.

We greatly appreciate the editor’s kind guidance and support.

**Action:** All funding-related text has been deleted from the manuscript, and the correct Funding Statement has been included in the cover letter.

**Comment #6**: Please amend your authorship list in your manuscript file to include author Jinsheng Zhao.

**Response:** We sincerely thank the editor for this reminder. We have carefully revised the manuscript and updated the authorship list to include Jinsheng Zhao as one of the co-authors.

**Action:** The authorship list in the revised manuscript file has been amended accordingly.

**Comment #7**: Please amend the manuscript submission data (via Edit Submission) to include author Jinfeng Li.

**Response:** We sincerely thank the editor for this helpful reminder. We have carefully revised the manuscript submission information in the online system and updated it to include Jinfeng Li as one of the co-authors.

**Action:** The submission data have been amended accordingly to include Jinfeng Li.

**Comment #8**: If the reviewer comments include a recommendation to cite specific previously published works, please review and evaluate these publications to determine whether they are relevant and should be cited. There is no requirement to cite these works unless the editor has indicated otherwise.

**Response:** We sincerely thank the editor for this reminder. We have carefully reviewed and evaluated the references suggested by the reviewers. Based on their relevance and contribution to our study, we have made appropriate judgments and incorporated citations where they benefit the revision of our manuscript.

We greatly appreciate the constructive suggestions and guidance from the reviewers and the editor.

**Re: The Itemized Response to Comments Made by Reviewer #1**

Journal:	**PLOS One**

Manuscript ID:	**PONE-D-25-35735**

Title:	**Study on crude oil displacement efficiency by fracturing fluid in tight sandstone reservoir**

Dear Editors and Reviewers:

Thank you for your letter and for the comments concerning our manuscript entitled “Study on crude oil displacement efficiency by fracturing fluid in tight sandstone reservoir” (ID: PONE-D-25-35735). Those comments are all valuable and very helpful for revising and improving our paper, as well as the important guiding significance to our researches. We have studied comments carefully and have made correction which we hope meet with approval. Revised portion are marked in red in the paper. The main corrections in the paper and the responds to the reviewer’s comments are as flowing:

This manuscript focuses on the study of the displacement efficiency of fracturing fluid on crude oil in tight sandstone reservoirs, conducting relevant experiments, and exhibiting certain innovativeness and practicality. However, there are some deficiencies need to be checked out and revised:

**Comment #1**: In Introduction section, author mentioned imbibition, but there is a lack of the relevance of imbibition to the main work of this manuscript. It is recommended that author should clarify this relevance to enhanced the readability of the manuscript.

**Response:** We sincerely thank the reviewer for this constructive suggestion. We agree that it is important to clarify the role of imbibition in relation to the main work of this manuscript. In the revised Introduction, we have added specific explanations to highlight that the displacement efficiency of fracturing fluids is primarily determined by the imbibition efficiency during the soaking stage. Specifically, during the soaking stage, the fracturing fluid spontaneously enters into the rock pores through the imbibition effect, displacing the crude oil within them. This process usually occurs between the fractures created by fracturing and the reservoir matrix. After the soaking stage, the displaced crude oil is produced together with the fracturing fluid during the flowback stage. In addition, we have supplemented the background description of imbibition in the Introduction to further clarify its importance in tight reservoirs. This addition strengthens the logical connection between the imbibition mechanism and the objectives of this study, demonstrating that imbibition plays a central role in evaluating the oil displacement efficiency of different fracturing fluids.

**Action:** The following statement was added to Introduction:

“During the soaking stage, the fracturing fluid spontaneously enters into the rock pores through the imbibition effect, displacing the crude oil within them. This process usually occurs between the fractures created by fracturing and the reservoir matrix. After the soaking stage, the displaced crude oil is produced together with the fracturing fluid during the flowback stage, forming an oil-water mixture that significantly increases crude oil production and reduces the flowback volume of fracturing fluid.”

“Imbibition is more common in low-permeability and tight reservoirs due to the presence of microscopic pore throats and fractures formed during hydraulic fracturing. Since the 1950s, imbibition has been extensively studied and developed as an important method of oil production.”

**Comment #2**: The relationship of imbibition and the flowback of fracturing fluid should be clarified in introduction (for example, the flowback of fracturing fluid may be achieved through imbibition).

**Response:** We thank the reviewer for this insightful comment. We agree that the relationship between imbibition and the flowback of fracturing fluid should be clarified in the Introduction. In the revised manuscript, we have highlighted that the soaking stage involves spontaneous imbibition of fracturing fluid into the rock pores, displacing crude oil that is subsequently produced during flowback. Furthermore, the flowback of fracturing fluid in tight reservoirs is closely related to the imbibition process, as imbibition not only displaces crude oil but also facilitates the return of fracturing fluid during the flowback stage.

**Action:** The following statement was added to Introduction:

“During the soaking stage, the fracturing fluid spontaneously enters into the rock pores through the imbibition effect, displacing the crude oil within them. This process usually occurs between the fractures created by fracturing and the reservoir matrix. After the soaking stage, the displaced crude oil is produced together with the fracturing fluid during the flowback stage.”

**Comment #3**: The case of letters in table 1 needs to be consistent.

**Response:** We sincerely thank the reviewer for pointing out this formatting issue. We have carefully checked Table 1 and revised the capitalization of all letters to ensure consistency throughout the table.

**Action:** The letter case in Table 1 has been standardized and corrected in the revised manuscript.

**Comment #4**: It is recommended that other physical parameters of experimental cores such as length, diameter and porosity should be shown in table 1.

**Response:** We sincerely thank the reviewer for this valuable suggestion. We agree that including additional physical parameters of the experimental cores will improve the completeness of the data presentation. Accordingly, we have revised Table 1 in the manuscript to add the length, diameter, and porosity of the experimental cores.

**Action:** Table 1 has been updated as follows:

**Table 1 Experimental material properties and experimental scheme**

|  | Core number | Length (cm) | Diameter (cm) | Porosity (%) | Permeability（10^-3^μm^2^） | Types of displacement fluid | Oil-water interfacial tension（mN/m) | Displacement time（h） |
| --- | --- | --- | --- | --- | --- | --- | --- | --- |
| 1 | | 3.400 | 2.520 | 5.83 | 0.121 | Guanidine gum gel-breaking fluid | 0.059 | 24 |
| 2 | | 3.400 | 2.520 | 5.83 | 0.121 | Cross-linked guanidine gum breaking fluid | 0.065 | 24 |
| 3 | | 2.615 | 2.520 | 8.62 | 0.110 | Slick water breaking fluid | 0.613 | 24 |
| 4 | | 2.615 | 2.520 | 8.62 | 0.110 | Slick water breaking fluid**+**0.3%OC-1 | 0.055 | 24 |
| 5 | | 4.980 | 2.520 | 7.31 | 0.149 | Guanidine gum gel-breaking fluid | 0.059 | 4,8,12,16 |

**Comment #5**: Is the method that converting the abscissa of the NMR T₂ spectrum to pore-throat radius commonly used in the industry? Author should appropriately cite some relevant literatures to demonstrate its rationality.

**Response:** We sincerely thank the reviewer for this insightful comment. We agree that it is important to clarify the rationality of converting the abscissa of the NMR T_2_ spectrum to pore-throat radius. This method has been widely used in tight oil and gas reservoir studies to characterize pore structure and fluid distribution. To address the reviewer’s suggestion, we have added relevant references to demonstrate its rationality and common use in the industry.

**Action:** The following statement was added to Experimental Section:

“The conversion of NMR T_2_ relaxation time to pore-throat radius is a widely accepted approach for pore structure characterization in tight reservoirs.”

**Reference:**

Zhu D, Li BF, Zheng L, Husein MM, Xu ZX, Li BL, et al. Experimental investigation into CO_2_ huff-n-puff in low-permeability heavy oil reservoirs: Role of fractures. Pet Sci. 2025; 22(3): 1200-1213.

Zhang X, Wei B, You JY, Liu J, Wang DL, Lu J, et al. Characterizing pore-level oil mobilization processes in unconventional reservoirs assisted by state-of-the-art nuclear magnetic resonance technique. Energy. 2021; 236: 121549.

**Comment #6**: Introduction section mentioned that the study revealed the main factors affecting oil-water displacement during the flowback process of tight oil fracturing, but the discussions of main factor sin Section 2 and 3 is not enough, which is confused.

**Response:** We sincerely thank the reviewer for this valuable suggestion. In our study, we discussed the effects of fracturing fluid viscosity, the addition of surfactants, and displacement time on displacement efficiency. However, we acknowledge that the analysis of these results was not sufficiently detailed in the original version. In the revised manuscript, we have supplemented the Experimental Results and Analysis Section with additional analysis of how fracturing fluid viscosity and surfactants influence displacement efficiency. These revisions ensure that the discussion of the main influencing factors is more comprehensive and consistent with the objectives stated in the Introduction. We greatly appreciate the reviewer’s constructive feedback.

**Action:** The following statement was added to Experimental Results and Analysis Section:

“The NMR results clearly show that after 24 hours of oil-water displacement, the signal amplitude of the cores is significantly lower than that of the oil-saturated cores, particularly in the regions corresponding to small and medium pores. This indicates that crude oil in these pores is preferentially displaced, leading to a marked reduction in oil content.”

“And low-viscosity fluids exhibit better mobility, enabling them to enter small and medium pores more effectively, establish stable flow pathways, and promote efficient displacement.”

“Therefore, viscosity is a key controlling factor for displacement efficiency. Lower-viscosity fracturing fluids achieve higher oil recovery by enhancing displacement in small and medium pores, whereas higher-viscosity fluids are less effective and may even contribute to oil redistribution into micropores. Hence, in the process of fracturing, special attention should be paid to the controlling effect of displacement fluid viscosity on oil recovery performance.”

“The addition of surfactants can greatly reduce the oil-water interfacial tension, which enhances the deformability of crude oil droplets, allowing them to flow smoothly through pore throats where they would otherwise be trapped under high interfacial tension conditions during the imbibition process. This promotes better crude oil mobility, enlarges the swept volume, and improves displacement efficiency. In addition, a lower interfacial tension can also reduce the adhesion work of crude oil, facilitating its detachment from the rock surface. And with the decreasing adhesion work, the oil-washing efficiency increases.”

**Comment #7**: The manuscript only reviewed the effect of capillary force on imbibition, while gravity also plays an important role in affecting imbibition. To better explain the background and relevant theories of this study, it is recommended to cite the following literatures:

a) https://doi.org/10.1016/j.geoen.2024.213071.

b) https://doi.org/10.2118/224403-PA.

c) https://doi.org/10.1080/01932691.2023.2177670.

d) https://doi.org/10.1016/j.geoen.2024.213057.

**Response:** We sincerely thank the reviewer for this valuable suggestion. We have carefully reviewed the relevant literature on the role of gravity in spontaneous imbibition and fully agree that gravity, together with capillary force, governs the displacement process. In the revised manuscript, we have modified the Introduction Section to define spontaneous imbibition as the process in which the wetting phase displaces the non-wetting phase fluid under the combined action of capillary force and gravity. We have also incorporated the recommended references to strengthen the theoretical background of this study.

**Action:** The following statement was added to Introduction Section:

“Spontaneous imbibition refers to the process in which the wetting phase displaces the non-wetting phase fluid from the porous medium under the combined action of capillary force and gravity.”

**Reference:**

Xie K, Wu ZQ, Liu CL, Mei J, Cao WJ, Ding HN, et al. A review of mechanisms and ML-based research on factors affecting spontaneous imbibition of surfactant. Geoenergy Sci Eng. 2024; 240: 213071.

Cao WJ, Qi YK, Xie K, Yan K, Lu XG, Bai MY, et al. Research on physical simulation model production and remaining oil distribution based on fluvial compound sand body architecture SPE J. 2024; 30(3): 1366-1376.

Yan X, Dai CL, Meng SW, Wei ZJ, Jin X, Liu H, et al. An efficient method for imbibition in asphaltene-adsorbed tight oil-wet reservoirs. Geoenergy Sci Eng. 2024; 240: 213057.

**Re: The Itemized Response to Comments Made by Reviewer #2**

Journal:	**PLOS One**

Manuscript ID:	**PONE-D-25-35735**

Title:	**Study on crude oil displacement efficiency by fracturing fluid in tight sandstone reservoir**

Dear Editors and Reviewers:

Thank you for your letter and for the comments concerning our manuscript entitled “Study on crude oil displacement efficiency by fracturing fluid in tight sandstone reservoir” (ID: PONE-D-25-35735). Those comments are all valuable and very helpful for revising and improving our paper, as well as the important guiding significance to our researches. We have studied comments carefully and have made correction which we hope meet with approval. Revised portion are marked in red in the paper. The main corrections in the paper and the responds to the reviewer’s comments are as flowing:

The paper is lacking with the several important results. The present form of the manuscript is not acceptable for possible publication in the journal. The comments that need to be addressed as follows:

**Comment #1**: While the authors have applied the tight sandstone method in their study, it would be beneficial to provide a brief introduction to this method for a clearer understanding by readers. Very few manuscripts have been considered to address the issues which is not sufficient.

**Response:** We sincerely thank the reviewer for this constructive suggestion. We agree that a clearer introduction to tight sandstone reservoirs will improve the readability and context of the manuscript. In the revised version, we have supplemented the beginning of the Introduction with a description of the characteristics of tight sandstone reservoirs, including the low permeability, nanoscale pore-throat structures, and geological complexity. We have also highlighted the current stage of EOR research, emphasizing the synergistic use of physical, chemical, and gas-flooding methods, and the practical application of imbibition and CO_2_ injection techniques. These additions provide readers with a more comprehensive understanding of the reservoir conditions that motivate this study.

**Action:** The following statement was added to Introduction Section:

“Tight sandstone reservoirs are characterized by low permeability, nanoscale pore-throat development, and poor reservoir properties, with significant geological complexity and engineering challenges. At present, research on enhanced oil recovery (EOR) in tight sandstone reservoirs has entered a new stage of “physical-chemical-gas flooding” synergistic development. Techniques such as imbibition after fracturing and CO_2_ injection (including both flooding and huff-n-puff methods) have been widely applied in practice.”

**Reference:**

Liu GF, Wang HL, Tang JC, Liu ZK, Yang DY. Effect of wettability on oil and water distribution and production performance in a tight sandstone reservoir. Fuel, 2023; 341: 127680. https://doi.org/10.1016/j.fuel.2023.127680.

**Comment #2**: The inclusion of Table 1, comparing results across various studies, is appreciated. However, the novelty of the study could be better emphasized. Strengthening the content related to the novelty would enhance the paper.

**Response:** Thank you very much for your valuable suggestion. We fully agree with your comment that the novelty of the study should be emphasized more clearly. According to your recommendation, we have carefully revised the Introduction section to better highlight the innovative aspects of this work.

Specifically, we have added a statement pointing out that the effects of fracturing fluid properties, such as fluid type and viscosity, on displacement efficiency have rarely been investigated. Furthermore, we emphasized that advanced nuclear magnetic resonance (NMR) technology is employed in this study to investigate pore-scale oil mobilization characteristics under different experimental conditions. This approach allows us to clarify the oil recovery behavior within various pore types under different influencing factors and to elucidate the mechanisms by which different fracturing fluid systems displace crude oil. Finally, we highlighted that the research results will provide not only theoretical and practical references for optimizing the post-fracturing flowback system, but also guidance for selecting appropriate fracturing fluid types in tight oil reservoirs.

We sincerely appreciate your insightful comments, which helped us improve the clarity and novelty of our manuscript.

**Action:** The following statement was added to Introduction Section:

“And the effects of fracturing fluid properties such as fracturing fluid type and viscosity on displacement efficiency have also been rarely investigated.”

“Meanwhile, advanced nuclear magnetic resonance (NMR) technology is employed to investigate the pore-scale oil mobilization characteristics of core samples under different experimental conditions, clarifying the oil recovery behavior within various types of pores under different influencing factors and elucidating the mechanisms by which different fracturing fluid systems displace crude oil. These research results will provide a strong theoretical and practical reference for optimizing the post-fracturing flowback system and guiding the selection of fracturing fluid types in tight oil reservoirs.”

**Comment #3**: Although the introduction section offers a comprehensive overview, it would be beneficial to include more recent works in the field to further contextualize the study.

**Response:** Thank you very much for your constructive suggestion. We agree with your comment that the Introduction section could benefit from the inclusion of more recent studies to better contextualize our work. According to your recommendation, we have carefully revised the Introduction and supplemented it with several up-to-date references related to oil-water displacement and imbibition in tight reservoirs.

**Action:** The following statements were added to Introduction Section:

“At present, research on enhanced oil recovery (EOR) in tight sandstone reservoirs has entered a new stage of “physical–chemical–gas flooding” synergistic development. Techniques such as imbibition after fracturing and CO_2_ injection (including both flooding and huff-n-puff methods) have been widely applied in practice.”

“Imbibition is more common in low-permeability and tight reservoirs due to the presence of microscopic pore throats and fractures formed during hydraulic fracturing. Since the 1950s, imbibition has been extensively studied and developed as an important method of oil production. Spontaneous imbibition refers to the process in which the wetting phase displaces the non-wetting phase fluid from the porous medium under the combined action of capillary force and gravity.”

“In addition, Zhu et al. conducted core imbibition experiments under different crude oil viscosities and core permeabilities, and the results showed that imbibition recovery increased with the decreasing oil viscosity and the increasing core permeability.”

**Reference:**

Liu GF, Wang HL, Tang JC, Liu ZK, Yang DY. Effect of wettability on oil and water distribution and production performance in a tight sandstone reservoir. Fuel, 2023; 341: 127680.

Zhu D, Li BF, Zheng L, Lei WS, Li BL, Li ZM. Effects of CO_2_ and surfactants on the interface characteristics and imbibition process in low-permeability heavy oil reservoirs. Colloids Surf., A. 2023; 657: 130538.

Xie K, Wu ZQ, Liu CL, Mei J, Cao WJ, Ding HN, et al. A review of mechanisms and ML-based research on factors affecting spontaneous imbibition of surfactant.	Geoenergy Sci Eng. 2024; 240: 213071.

Cao WJ, Qi YK, Xie K, Yan K, Lu XG, Bai MY, et al. Research on physical simulation model production and remaining oil distribution based on fluvial compound sand body architecture SPE J. 2024; 30(3): 1366-1376.

Yan X, Dai CL, Meng SW, Wei ZJ, Jin X, Liu H, et al. An efficient method for imbibition in asphaltene-adsorbed tight oil-wet reservoirs. Geoenergy Sci Eng. 2024; 240: 213057.

**Comment #4**: Full methodology section is incomplete such as sample preparation, testing and error analysis.

**Response:** Thank you very much for your helpful suggestion. We agree with your observation that the methodology section in the original version was incomplete. In the revised manuscript, we have made corresponding improvements by adding the physical parameters of the core samples (length, diameter, and porosity) in Table 1, refining the experimental procedures to provide clearer descriptions of core preparation and testing steps, and supplementing the NMR testing details, including the instrument information and specific parameters used in this study. These revisions ensure that the methodology section is now more comprehensive, rigorous, and reproducible. We sincerely appreciate your constructive advice, which has significantly improved the clarity and quality of our manuscript.

**Action:** The following statements were added to the Experimental Section:

“(1) After testing the porosity and permeability of the natural sandstone cores, the cores were dried, vacuumed, and then saturated under high pressure with formation water containing 25000 mg/L of MnCl_2_.”

“(4) Under simulated reservoir temperature conditions, the prepared cores were immersed in different types of fracturing fluids. According to the experimental design shown in Table 1, the type of working fluid, the proportion of additives, and the soaking time were varied. The overall experimental procedure is illustrated in Figure 1.”

“In this study, a MesoMR23-060 NMR imaging system (Suzhou Niumag Analytical Instrument Corporation, Suzhou, China) was used for core testing to investigate the distribution of crude oil within the core samples. This equipment is equipped with an independent pulse control module and a radio frequency (RF) transmission/reception circuit, which improves the accuracy and stability of pulse sequences and enhances the signal-to-noise ratio of NMR signals. The spectrometer frequency of the RF pulse used in this study was 21 MHz, and the waiting time between two scans of sampling data was 4000 ms.”

**Comment #5**: Authors could add more explanation for this statement. 'Fig. 4 - Fig. 6 were sketched to portrayed the streamline graphs for the signal amplitude problems under discussion for distinct values of pores. They stated that increasing in the pores, the graphs of streamline more presiding.'

**Response:** Thank you very much for your valuable suggestion. We agree that the explanation of Figures 4-6 and the description of the NMR T_2_ spectrum required further clarification. In the revised manuscript, we have added an explanation stating that the vertical signal amplitude represents the crude oil content within pores of the corresponding size, and that a decrease in signal amplitude after the displacement experiment indicates that crude oil in those pores has been displaced and produced. This addition clarifies the meaning of the streamline graphs and strengthens the link between the NMR results and the pore-scale displacement process.

In addition, to further support the rationality of converting NMR T_2_ relaxation time into pore-throat radius, we have supplemented the Experimental Section with a reference to relevant literature, highlighting that this conversion method is a widely accepted approach for pore structure characterization in tight reservoirs.

We sincerely appreciate your constructive comments, which have helped us improve the clarity, rigor, and completeness of our manuscript.

**Action:** The following statements were added to the Experimental Section:

“The signal amplitude on the vertical axis represents the crude oil content within pores of the corresponding size, and a decrease in signal amplitude after the experiment indicates that crude oil in those pores has been displaced and produced.”

“The conversion of NMR T_2_ relaxation time to pore-throat radius is a widely accepted approach for pore structure characterization in tight reservoirs.”

**Reference:**

Zhu D, Li BF, Zheng L, Husein MM, Xu ZX, Li BL, et al. Experimental investigation into CO_2_ huff-n-puff in low-permeability heavy oil reservoirs: Role of fractures. Pet Sci. 2025; 22(3): 1200-1213.

Zhang X, Wei B, You JY, Liu J, Wang DL, Lu J, et al. Characterizing pore-level oil mobilization processes in unconventional reservoirs assisted by state-of-the-art nuclear magnetic resonance technique. Energy. 2021; 236: 121549.

**Comment #6**: Many of the study's results are presented without thorough explanation. Adding citations in the results and discussion section would help properly convey the significance of each finding and provide a more comprehensive analysis.

**Response:** Thank you very much for your constructive suggestion. We agree with your observation that the results and discussion section in the original version lacked sufficient explanation and supporting references. To address this issue, we have revised the manuscript by expanding the analysis of the experimental results and supplementing them with relevant citations.

Specifically, we have clarified that the NMR results clearly show a significant decrease in signal amplitude after 24 hours of oil-water displacement, particularly in small and medium pores, which indicates preferential crude oil displacement from these pore types. We further explained that low-viscosity fracturing fluids exhibit better mobility, allowing them to penetrate small and medium pores more effectively, thereby enhancing oil recovery, whereas high-viscosity fluids are less effective and may even lead to oil redistribution into micropores. In addition, we have expanded the discussion of surfactants, explaining that the addition of surfactants significantly reduces oil-water interfacial tension, enhances crude oil droplet deformability, enlarges the swept volume, reduces adhesion work, and thus improves the oil recovery. Relevant references have been added to support these explanations.

These revisions strengthen the interpretation of our results, place them in the context of existing research, and improve the comprehensiveness and academic depth of the results and discussion section. We sincerely appreciate your helpful advice, which has substantially enhanced the quality and clarity of our manuscript.

**Action:** The following statement was added to Experimental Results and Analysis Section:

“The NMR results clearly show that after 24 hours of oil-water displacement, the signal amplitude of the cores is significantly lower than that of the oil-saturated cores, particularly in the regions corresponding to small and medium pores. This indicates that crude oil in these pores is preferentially displaced, leading to a marked reduction in oil content.”

“And low-viscosity fluids exhibit better mobility, enabling them to enter small and medium pores more effectively, establish stable flow pathways, and promote efficient displacement.”

“Therefore, viscosity is a key controlling factor for displacement efficiency. Lower-viscosity fracturing fluids achieve higher oil recovery by enhancing displacement in small and medium pores, whereas higher-viscosity fluids are less effective and may even contribute to oil redistribution into micropores. Hence, in the process of fracturing, special attention should be paid to the controlling effect of displacement fluid viscosity on oil recovery performance.”

“The addition of surfactants can greatly reduce the oil-water interfacial tension, which enhances the deformability of crude oil droplets, allowing them to flow smoothly through pore throats where they would otherwise be trapped under high interfacial tension conditions during the imbibition process. This promotes better crude oil mobility, enlarges the swept volume, and improves displacement efficiency. In addition, a lower interfacial tension can also reduce the adhesion work of crude oil, facilitating its detachment from the rock surface. And with the decreasing adhesion work, the oil-washing efficiency increases.”

**Reference:**

Zhu D, Li BF, Zheng L, Lei WS, Li BL, Li ZM. Effects of CO_2_ and surfactants on the interface characteristics and imbibition process in low-permeability heavy oil reservoirs. Colloids Surf., A. 2023; 657: 130538.

**Comment #7**: Several minor typographical errors are present in the paper. Correcting these errors is essential for improved readability and credibility.

**Response:** Thank you very much for your careful review and valuable suggestion. We carefully reviewed the entire manuscript and corrected all the typographical, grammatical, and expression errors to improve readability and professionalism. For example, the sentence “There usually have a long backflow period for tight sand reservoirs after fracturing, which affects the production of oil wells.” was revised to “Tight sandstone reservoirs usually experience a long flowback period after hydraulic fracturing, which significantly affects oil production.” Similarly, the sentence “After fracturing, it is usually produced by the well soak to control fracturing fluid flowback,” was revised to “After fracturing, the well-soaking is commonly employed to control fracturing fluid flowback and enhance oil recovery.” We have made similar careful corrections throughout the manuscript, and all modifications are highlighted in the revised version with track changes for your convenience. We sincerely appreciate your meticulous attention, which has greatly helped us enhance the clarity and quality of our manuscript.

**Comment #8**: The axes labels and tick labels in the figures are too small, affecting clarity. Enhancing the font sizes of these labels would significantly improve readability.

**Response:** Thank you for your constructive suggestion. We fully agree that the clarity of figures is essential for improving the readability of the manuscript. Accordingly, we have carefully revised all figures by enlarging the font sizes of the axis labels, tick labels, and legends to ensure consistency and clear visibility. These adjustments significantly enhance the clarity and readability of the figures. The revised figures are included in the updated manuscript for your review.

We sincerely appreciate your valuable feedback, which has helped us improve the overall presentation quality of our work.

**Comment #9**: The conclusion section is not thoroughly presented.

**Response:** Thank you very much for your constructive suggestion regarding the conclusion section. We agree with your assessment that the original conclusion was not thoroughly presented. To address this, we have revised and expanded the conclusion section in the revised manuscript.

Specifically, we added an opening paragraph to clearly state the purpose and scope of this study: “In this study, different types of fracturing fluids are selected to conduct oil-water displacement experiments on tight sandstone cores. Combined with NMR testing, the pore-scale oil mobilization characteristics are clarified, and the effects of surfactant addition and displacement conditions on displacement efficiency are identified. These findings provide theoretical and practical guidance for optimizing post-fracturing flowback systems and selecting appropriate fracturing fluid types in tight oil reservoirs. The following conclusions can be drawn from this study.”

Furthermore, we revised the second conclusion to highlight the influence of fluid viscosity on displacement efficiency: “(2) The displacement efficiency of the low-viscosity gel-breaking fluid is 4.94% higher than that of the high-viscosity fluid. Low-viscosity fluids exhibit better mobility, enabling them to enter small and medium pores more effectively, establish stable flow pathways, and promote efficient displacement.”

In addition, we added a new conclusion to emphasize the effect of surfactants: “(3) The displacement efficiency of the gel-breaking fluid containing surfactant is 3.62% higher than that of the fluid without surfactant. By significantly reducing oil-water interfacial tension, the addition of surfactants enhances the deformability and mobility of crude oil droplets, thereby enlarging the swept volume and improving displacement efficiency.”

These modifications provide a more comprehensive summary of the key findings and mechanisms identified in this study, thereby strengthening the completeness and readability of the conclusion section. We sincerely appreciate your helpful comments, which allowed us to improve the manuscript substantially.

**Action:** The following statement was added to Conclusions Section:

“In this study, different types of fracturing fluids are selected to conduct oil-water displacement experiments on tight sandstone cores. Combined with NMR testing, the pore-scale oil mobilization characteristics are clarified, and the effects of surfactant addition and displacement conditions on displacement efficiency are identified. These findings provide theoretical and practical guidance for optimizing post-fracturing flowback systems and selecting appropriate fracturing fluid types in tight oil reservoirs. The following conclusions can be drawn from this study.”

“(2) The displacement efficiency of the low-viscosity gel-breaking fluid is 4.94% higher than that of the high-viscosity fluid. Low-viscosity fluids exhibit better mobility, enabling them to enter small and medium pores more effectively, establish stable flow pathways, and promote efficient displacement.

(3) The displacement efficiency of the gel-breaking fluid containing surfactant is 3.62% higher than that of the fluid without surfactant. By significantly reducing oil-water interfacial tension, the addition of surfactants enhances the deformability and mobility of crude oil droplets, thereby enlarging the swept volume and improving displacement efficiency.”

**Re: The Itemized Response to Comments Made by Reviewer #3**

Journal:	**PLOS One**

Manuscript ID:	**PONE-D-25-35735**

Title:	**Study on crude oil displacement efficiency by fracturing fluid in tight sandstone reservoir**

Dear Editors and Reviewers:

Thank you for your letter and for the comments concerning our manuscript entitled “Study on crude oil displacement efficiency by fracturing fluid in tight sandstone reservoir” (ID: PONE-D-25-35735). Those comments are all valuable and very helpful for revising and improving our paper, as well as the important guiding significance to our researches. We have studied comments carefully and have made correction which we hope meet with approval. Revised portion are marked in red in the paper. The main corrections in the paper and the responds to the reviewer’s comments are as flowing:

Hello, Author. Thank you for submitting this manuscript for review. I want to commend the thoroughness of the research; however, I have highlighted its strengths and some areas where it could be improved.

**Comment #1**: This study addresses relevant practical problems like long flowback in tight reservoirs. It employs suitable experimental methods such as core flooding and NMR T_2_ analysis to examine the displacement efficiency of different fracturing fluid gel-breakers. The experimental design controls key variables such as permeability through core pairs, interfacial tension, and viscosity to isolate their effects. Using NMR for pore-level displacement measurement is a strength. The conclusions about pore size impact, viscosity, interfacial tension, and time dependency are reasonable extensions of the experimental data.

Tables 2, 3, and 4 and Figures 4, 5, and 6 generally support the main conclusions regarding the effects of viscosity (Table 2), surfactants/interfacial tension (Table 3), and displacement time (Table 4) on overall and pore-specific displacement efficiency. The data support the observation of negative efficiency in micropores and plausibly explained.

However, the main weakness is the lack of replication. The study uses only one core per condition (e.g., one core for guanidine gum, one for cross-linked gum, one for slickwater, one for slickwater+surfactant). Although cores from longer samples were used for some comparisons (cores 1-2, 3-4), this does not constitute true replication. Natural heterogeneity within a single long core means results from one sample may not be representative. Without replicates, the reliability and general applicability of the efficiency values (e.g., 23.25% vs 18.31%, 13.11% vs 16.73%) are significantly reduced. While the directions of effects (e.g., lower viscosity increases efficiency) are probably valid due to controlled comparisons, the effect magnitudes and absolute efficiency values lack solid statistical support since n=1 per condition. The time-dependent study (Core 5) also involved only one core sequentially.

**Response:** Thank you very much for your valuable comments. We fully agree with your observation that the lack of replication is a limitation of our study. We sincerely apologize for this shortcoming.

Due to the inherent heterogeneity of natural tight sandstone cores, the pore structures of each core are never identical. Therefore, even under the same experimental conditions, it is not possible to obtain completely identical results. As a result, our experiments aimed to reveal general trends rather than absolute values. To minimize the effect of heterogeneity, we cut paired samples from the same long core whenever possible, ensuring similar pore-throat structures and permeability.

In addition, for the calculation of recovery efficiency, we used nuclear magnetic resonance (NMR) testing to ensure the accuracy of crude oil production and oil recovery data. NMR allows us to directly detect the crude oil content in cores. We have supplemented the manuscript with a description of the underlying principle, as well as detailed information about the instrument and parameters to ensure reproducibility of testing.

We acknowledge that the absence of true replication remains a limitation of this study, and we will further explore more advanced experimental approaches and statistical analyses in future work to enhance the robustness and general applicability of our findings.

We sincerely appreciate your insightful suggestions, which have helped us improve both the quality and transparency of our manuscript.

**Action:** The following statement was added to Experimental Section:

“In this study, a MesoMR23-060 NMR imaging system (Suzhou Niumag Analytical Instrument Corporation, Suzhou, China) was used for core testing to investigate the distribution of crude oil within the core samples. This equipment is equipped with an independent pulse control module and a radio frequency (RF) transmission/reception circuit, which improves the accuracy and stability of pulse sequences and enhances the signal-to-noise ratio of NMR signals. The spectrometer frequency of the RF pulse used in this study was 21 MHz, and the waiting time between two scans of sampling data was 4000 ms.”

**Comment #2**: The manuscript reports no statistical analysis at all. Results are presented as single measurements (e.g., displacement efficiencies in tables) without any variance measures (standard deviation, error bars), significance tests (t-tests, ANOVA), or discussion of uncertainty. Given that only one core per condition was used (n=1), rigorous statistical comparison of treatments is impossible. The lack of acknowledgment of this limitation or attempts to quantify uncertainty (such as repeated measurements if feasible) means the analysis is not rigorous or appropriate.

**Response:** We sincerely thank you for your valuable comment and apologize for this limitation in our study. As you pointed out, statistical analysis is not possible in our work since each experimental condition was conducted with only one natural tight sandstone core. Due to the strong heterogeneity of natural cores, even samples with similar porosity and permeability can still differ significantly in their pore structures. This makes it extremely difficult to obtain multiple cores with comparable pore structures for repeated experiments. To minimize the effect of heterogeneity, we cut longer cores into paired shorter samples for comparative testing. However, if we continued cutting cores further to create replicates, the sample scale would become too small and the experimental results would introduce substantial errors.

In response to your suggestion, we have revised the results and discussion section to explicitly acknowledge this limitation. We have clarified that since each experiment was performed only once, replication and statistical testing were not possible. Therefore, the displacement efficiency values presented in this study should be regarded as reflecting general trends rather than statistically validated absolute values.

We acknowledge this shortcoming and will explore more advanced experimental approaches and statistical methods in future studies to improve the robustness of our findings. Once again, we sincerely thank you for your constructive suggestion, which has helped us enhance the rigor and transparency of the manuscript.

**Comment #3**: The manuscript does not specify that raw data (like NMR T_2_ spectra, core properties beyond permeability, fluid measurements) have been deposited in a public repository or provided as supplementary material. While summary results are shown in figures and tables, raw data necessary for reproduction or re-analysis are not explicitly available in the manuscript or declared as accessible elsewhere.

**Response:** Thank you very much for your constructive suggestion. In response, we have supplemented the physical properties of the core samples beyond permeability, including length, diameter, and porosity, which are now shown in Table 1. In addition, all original NMR T_2_ spectra, as well as the NMR and mercury intrusion test data, have been provided in the Supporting Information files to ensure reproducibility and transparency of the study.

We sincerely appreciate your helpful comment, which has allowed us to improve the completeness and accessibility of our data.

**Action:** The revised version of Table 1 is provided as follows:

**Table 1 Experimental material properties and experimental scheme**

|  | Core number | Length (cm) | Diameter (cm) | Porosity (%) | Permeability（10^-3^μm^2^） | Types of displacement fluid | Oil-water interfacial tension（mN/m) | Displacement time（h） |
| --- | --- | --- | --- | --- | --- | --- | --- | --- |
| 1 | | 3.400 | 2.520 | 5.83 | 0.121 | Guanidine gum gel-breaking fluid | 0.059 | 24 |
| 2 | | 3.400 | 2.520 | 5.83 | 0.121 | Cross-linked guanidine gum breaking fluid | 0.065 | 24 |
| 3 | | 2.615 | 2.520 | 8.62 | 0.110 | Slick water breaking fluid | 0.613 | 24 |
| 4 | | 2.615 | 2.520 | 8.62 | 0.110 | Slick water breaking fluid**+**0.3%OC-1 | 0.055 | 24 |
| 5 | | 4.980 | 2.520 | 7.31 | 0.149 | Guanidine gum gel-breaking fluid | 0.059 | 4,8,12,16 |

All other data presented in the manuscript are provided in the Supporting Information.

**Comment #4**: The manuscript is generally clear and follows the conventional structure (Abstract, Intro, Methods, Results, Discussion, Conclusion). Technical terminology is appropriate. However, there are some instances of non-standard English and grammatical errors that slightly hinder readability but do not obscure the meaning:

a. "There usually have a long backflow period" (Abstract - should be "is").

b. "produced by the well soak" (Abstract - phrasing is awkward).

c. "reservoir reservoirs" (Abstract - redundant)

d. Inconsistent capitalization (e.g., "Guanidine gum" vs "guanidine gum").

e. Sentences are often lengthy and would benefit from concise rewriting and professional copyediting.

**Response:** Thank you very much for your valuable feedback on the English usage and grammar in our manuscript. We have carefully revised the manuscript to address the issues you highlighted:

a. The sentence “There usually have a long backflow period” in the Abstract has been revised to: “Tight sandstone reservoirs usually experience a long flowback period after hydraulic fracturing, which significantly affects oil production.”

b. The phrase “produced by the well soak” has been rephrased as: “After fracturing, the well-soaking is commonly employed to control fracturing fluid flowback and enhance oil recovery.”

c. The redundant phrase “reservoir reservoirs” has been corrected to “tight sandstone reservoirs.”

d. We carefully checked capitalization throughout the manuscript to ensure consistency, for example, standardizing “guanidine gum.”

e. Several lengthy sentences have been revised into simpler, more concise forms to improve readability and clarity. These modifications are highlighted in the revised manuscript (marked in red).

In addition, we have thoroughly re-checked the entire manuscript to correct any remaining language errors and ensure overall clarity and consistency.

We sincerely appreciate your constructive comments, which have helped us improve the quality, readability, and professionalism of our manuscript.
